# A novel video compendium of real surgical patient interactions for medical students

**DOI:** 10.1016/j.sopen.2023.07.017

**Published:** 2023-07-27

**Authors:** Stephen J. O'Brien, Michelle Reardon, Gerald McGreal

**Affiliations:** Department of Surgery, Mercy University Hospital, Grenville Place, Cork, Ireland; Department of Surgery, School of Medicine, University College Cork, Ireland

**Keywords:** Surgical education, e-Learning, Medical student, COVID-19

## Abstract

**Objective:**

To develop a novel video compendium of real surgical patient interactions as a tool for medical student education and to evaluate our institutional experience of its usefulness.

**Design:**

*Prospective development of a video* compendium of real surgical patient interactions.

**Setting:**

Single university affiliated hospital in Cork, Ireland.

**Participants:**

Patients with illnesses relevant to the surgery curriculum and students from an Irish medical school.

**Results:**

Videos were recorded of the clinical interaction between a consultant surgeon and patients, capturing focused history taking and/or clinical examination, with an associated set of explanatory notes. Fifty videos were developed with a tiered release to the clinical year medical students, via their virtual learning/education platform. Three hundred and eleven students responded to the questionnaire across 3-student year groups (311/585–53 %). Fifty-two percent of students did not have their clinical rotations affected by the COVID-19 pandemic. >90 % of students agreed that the videos helped history taking and clinical examination technique. >80 % of students agreed that the accompanying text slides reinforced key points and helped with understanding difficult topics. Eighty-five percent of students reported that the videos increased exposure to surgical patients and pathology. Eighty-five percent of students rated their experience as at least 4 out of 5.

**Conclusions:**

This online educational compendium bridged a gap for students with limited clinical exposure during the COVID-19 pandemic, and has become an important resource for all clinical students. Our novel engagement with real patients sets this compendium apart from resources which use actors.

## Highlights

• This manuscript described the development of a novel video compendium of real patient surgical interactions.
• This was complimented by a series of accompanying text slides to expand on the video content.
• By using a questionnaire, the majority of students expressed a highly positive experience with the videos and text slides.

## Introduction

Surgical education is a core component of the curriculum for medical students. Developing the skill set to identify, investigate, and treat surgical pathology is important for doctors at all levels of training. Significant effort is required on behalf of both medical school lecturers and students to achieve the standards required for contemporary healthcare. The cumulative exposure to surgical patients during medical school can be limited. This is due in part to surgical education being restricted to the clinical years, and to the competing demands of the academic calendar.

COVID-19 has had a marked secondary effect on the delivery of medical school education and the experience of medicals students since 2020 [1,2]. The greatest impact was In January and February 2021 when medical education was moved completely to online resources for 5–8 weeks, due to COVID restrictions [3], with minimal compensatory hospital placement added to the end of term in April/May. There have been many other interruptions to normal clinical practice due to intermittent reductions in the delivery of elective surgical care. When these circumstances are paired with student absences, or indeed consultant absences, associated with isolation periods due to COVID infection/contact, the net effect is that there has been a significant reduction in the exposure of medical students to the treatment and management of surgical patients throughout the academic years 2019/2020, 2020/2021 and 2021/2022.

It was our impression that student's clinical examination skills were declining in the years preceding COVID, though grade inflation suggested the opposite. While there are innumerable online resources for medical students to complement their education, these are often in the form of simulated patients, without real clinical findings. To augment clinical experience for students, we developed a novel compendium of real surgical patient interactions. The aim of this study is to describe our creation of surgical educational videos and to evaluate the experience of students who studied them.

## Methods

### Ethics statement

This study was approved by the Clinical Research Ethics Committee of the Cork Teaching Hospitals.

### Patients recruitment and video development

The first patients recruited for video recordings were those who attended the authors' hospital to facilitate the final year medical student short-case examinations in spring 2019. These patients had obvious clinical signs spanning a range of common surgical conditions. Subsequent patients were prospectively identified on an ad hoc basis between 2019 and 2021 in the outpatient clinic and on the surgical wards by the consultant surgeon or clinical lecturer. Patients were considered for inclusion if they described interesting symptoms, and/or had abnormal physical findings demonstrated by clinical examination, which are relevant to the curriculum. The patients selected were on the basis of the University College Cork curriculum (https://ucc-ie-public.courseleaf.com/modules/?details&code=cp5100). In the Mercy hospital, there full spectrum exposure to general surgery and vascular surgery pathology.

Patients gave written informed consent for participation in the study.

A video was recorded of the encounter between the consultant surgeon and the enrolled patient. This consisted of the consultant taking a history and/or performing a focused clinical examination. In the initial part of the series the video recording was performed using university audiovisual equipment. Subsequently, to offer greater flexibility, videos were recorded with a mobile phone device. This resulted in a slight deterioration in quality, including noise pollution in the ward setting. The videos were saved and stored on a university server and subsequently published on the teaching portal, Canvas. An accompanying set of notes for each video was developed. Each of the videos was also stored on a password protected university computer. Accompanying tutorials and quizzes were developed for some of the topics over the course of the year.

### Student cohort

In University College Cork, direct entry medical students undertake a 5-year program and graduate entry medical students undertake a 4-year program. Videos were released in a tiered fashion to 3rd-5th year direct entry students (DEM) and 3rd-4th year graduate entry students (GEM), through the university e-learning platform (Canvas). Videos capturing basic, common surgical conditions were made available to junior clinical students. More complex vascular content was released to students in their penultimate year, to correspond with learning objectives at that stage of the curriculum, while more complex gastro-intestinal content was reserved for final year students.

### Questionnaire development and pilot

As part of a quality improvement initiative for the video compendium, a follow up questionnaire was developed. This was piloted with 258 students at the end of the 2019/20 academic year. The questionnaire was revised (Supplementary Table 1). The finalized questionnaire was developed into a “Microsoft Form” document, and was sent at the end of the 2020/21 academic year to the university email address of each student in the year groups. The questionnaire responses were anonymised at the point of data entry by each student. The results of these questionnaires were exported to a Microsoft Excel spreadsheet.

### Statistical analysis

All statistical analysis was done using SPSS v26.0 (IBM Corp, Armonk NY). Categorical data was reported as median and interquartile range. Continuous data was reported as number and frequency. Questionnaire results were reported on a 5-point Likert scale (Strongly disagree, disagree, neutral, agree, strongly agree).

## Results

Fifty videos were developed over a 3-year period, encompassing general surgery, gastrointestinal surgery, and vascular surgery. The videos and accompanying text slides were organized into topics according to the surgery curriculum. The list of topics/ videos is shown in Table 1. There were 16 videos released to the DEM3/GEM2 students, 35 videos released to the DEM4/GEM3, and 40 videos released to the DEM5/GEM4 students. The average length of all videos was 8 min (Gastrointestinal surgery 8.5 min, vascular surgery 10 min, miscellaneous 6 min).

**Table 1:** Topics described in the video compendium by surgical specialty.

Clinical area	Recorded tutorials	Patient based videos
Basic principles and general surgery		
	Definitions	Lipoma
	Pre-op work-up	Cervical Lymph node examination
	Surgical Prophylaxis	Axillary & Inguinal LN examination
	Post-op instructions	Lower limb cellulitis
	Post-op Pyrexia	
	Wound Infection	
	Paralytic ileus	
	Post-op urinary retention	
	Fluid management & Nutrition	
	Venous access	
	Groin discomfort & lump	Clinical examination of patient with incisional hernia
		Inguinal Hernia Colorectal cancer metastasis
		Inguino-scrotal hernia
	Abdominal pain	Acute appendicitis
	Drains	
	Incisions/resections	
Upper gastrointestinal surgery		
	Dysphagia	Minimally invasive oesophagectomy for Cancer
	Upper Gastrointestinal tract bleed	Upper Gastrointestinal tract bleed & Goitre
		Peptic Ulcer Disease
Hepatobiliary and pancreatic surgery		
	Jaundice	Murphy's positive patient
	Hepato-biliary & pancreatic surgery introduction	Gallstone pancreatitis
	Hepato-biliary & pancreatic surgery signs & quiz	Clinical examination of jaundiced patient
	Principles of cancer surgery	
	Liver surgery	
Lower gastrointestinal surgery		
	Colorectal cancer	Hartmann's Procedure
		Rectal cancer with liver metastasis
	Stoma	Patient with Colostomy & ileal conduit
	Small bowel obstruction Abdominal distension	Small bowel obstruction history & clinical examination
	Lower abdominal pain	Large bowel obstruction & bladder cancer Diverticulitis history
		Diverticular fistula
		Large bowel obstruction & bladder cancer Diverticulitis history
	Lower gastrointestinal tract bleed	
Vascular surgery		
	Clinical examination	Foot pulses
	Peripheral vascular occlusive disease	Necrotic toe History & clinical examination
		Rest pain & tissue loss
		Axillo-bifemoral bypass- post operative
		Below knee amputation & carotid disease resulting in drop attacks
	Acute limb ischemia	Post-operative exam
		Right femoral-popliteal bypass for acute-on-chronic limb ischemia
		Acute upper limb ischaemia & thrombophilia
	Diabetic vascular disease	Diabetic vascular disease & clinical examination
		Diabetic foot infection clinical examination
		Diabetic foot ulcers & Charcot's deformity
		Young patient diabetic mellitus foot examination
		Charcot's foot & skin graft
	Venous hypertension	Venous hypertensive disease leg ulcer examination
		Venous ulcer & skin graft
	Abdominal aortic aneurysm	Symptomatic abdominal aortic aneurysm & radiology
		Ruptured abdominal aortic aneurysm & radiology
		Ruptured iliac artery aneurysm
		Endovascular aneurysm repair history, examination & radiology Bladder Cancer
	Carotid disease	Amaurosis Fugax
		Carotid endarterectomy- cerebro-vascular accident in a male with motor & speech deficit
		Carotid Endarterectomy- cerebro-vascular accident in a female with right-sided motor deficit
		Transient ischaemic attack- sensory & speech loss
	Miscellaneous	Chronic mesenteric Ischaemia
		Raynaud's disease
		Lumbar sympathectomy for neuropathic pain
		Haematoma & crush injury
		Abdominal examination in renal transplant patient

Three hundred and eleven students responded to the questionnaire across 3-student year groups (311/585–53 %) (Table 2). This comprised of 103 3rd DEM/2nd GEM students (50 %- 107/207), 89 4th DEM/3rd GEM students (52 %- 89/172), and 119 final med students (58 %- 119/206). The majority of respondents were female (61 %). Only 52 % of students did not have their placement affected by the COVID19 pandemic, with 36 % of students missing 2 or more weeks of a placement.

**Table 2:** Demographics of the student cohort that responded to the questionnaire.

Variable	N = 311 N(%)
Age, median (Interquartile range)	24 (23–26)
Gender	
Female	186 (61)
Male	119 (38)
Prefer not to say	4 (1)
Program	
DEM	183 (59)
GEM	126 (41)
Number of Weeks of surgical placement missed due to COVID	
No placement missed	163 (52)
1 week missed	32 (11)
2 weeks missed	81 (26)
>2 weeks missed	34 (11)
AV Issues with the videos	
Yes	36 (12)
No	251 (88)

*Number of videos watched*	
Year 3 (N = 103)	
<3	2 (2)
3–6	6 (6)
7–10	21 (21)
>10	72 (71)
Year 4 (N = 89)	
<9	24 (27)
9–18	27 (30)
18–27	21 (24)
27–35	17 (19)
Year 5 (119)	
<10	19 (16)
10–20	32 (27)
20–30	42 (36)
30–40	25 (21)

Data expressed as number (percentage) unless otherwise stated.

**Fig. 1:**
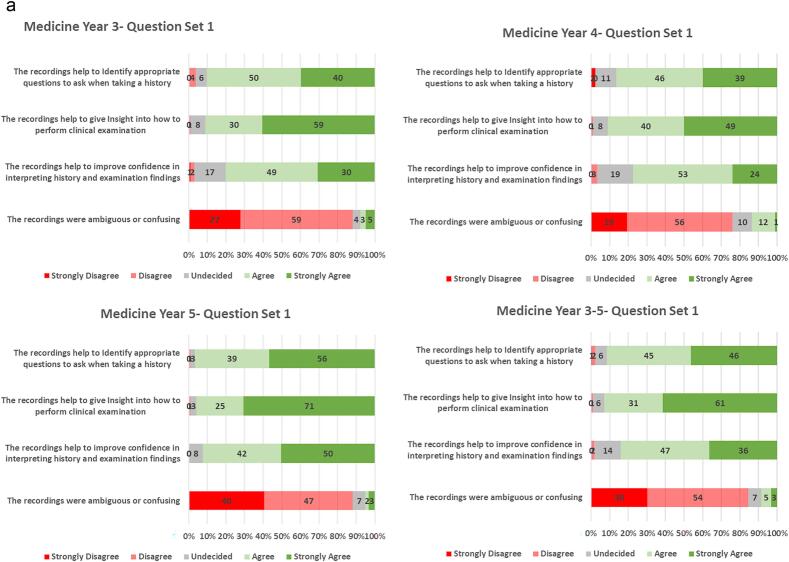

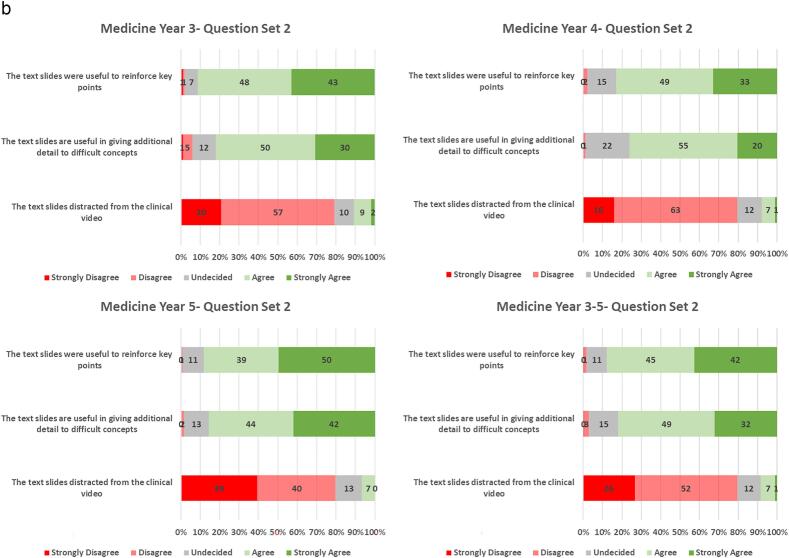

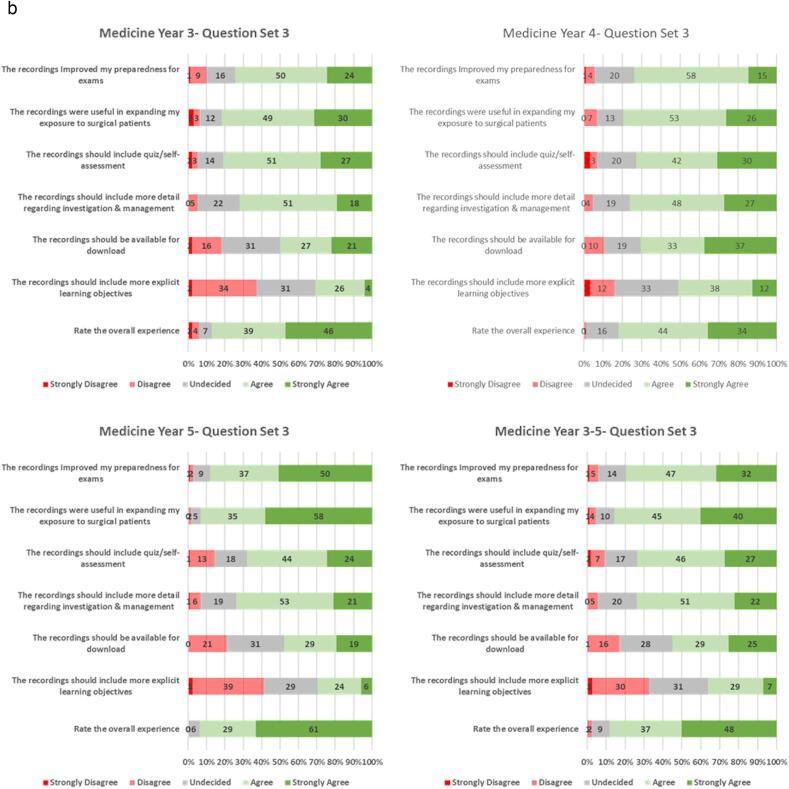
a. Description of questionnaire results, related to the patient recordings, on a 5-point Likert scale and stratified by student year group. Across all year groups the videos were beneficial in history taking and clinical examination skills. b. Description of questionnaire results, related to the accompanying text slides, on a 5-point Likert scale and stratified by student year group. The text slides were useful to students to reinforce and assist in the explanation of the video material. c. Description of questionnaire results, related to the usefulness of the videos for examinations overall experience. The results are presented on a 5-point Likert scale and stratified by student year group. Eighty-five percent of the overall student group rated the videos at least 4 out of 5.

>90 % of students agreed that the videos helped history taking and clinical examination technique. A small proportion (8 %) of the students agreed or strongly agreed that the videos were either ambiguous or confusing (Fig. 1a). To enhance the video compendium, an accompanying set of notes were developed. >80 % of students agreed or strongly agreed that the notes were useful to reinforce key points, and to give additional detail regarding difficult concepts. <8 % of students found the text slides distracted from the clinical video (Fig. 1b). Student use of tutorials and quizzes are shown in Supplementary Table 2. The Gastrointestinal Tract surgery tutorial was the most used among both 4th DEM/3rd GEM (31 %- 53/172) and final med students (52 %- 107/206). The Peri-operative care quiz was the most used among both 4th DEM/3rd GEM (46 %- 79/172) and final med students (14 %- 29/206).

Among all student groups, the majority agreed or strongly agreed that the videos helped with preparation for clinical examinations, and with exposure to surgical patients (Fig. 1c). As a point of critique, students did express an interest for the topics to contain quizzes or a self-assessment component (73 %). Consequently, some quizzes have subsequently been added to the package of resources. Students also agreed or strongly agreed (73 %) that the videos should describe more detail regarding investigation and management of the video topics. To avoid overcrowding the notes that accompany video recordings of patient interactions, a parallel set of recorded tutorials were created to give background detail including anatomy, physiology, investigations and treatment. In contrast, students did not think that more explicit learning objectives needed to be included. Interestingly, only a small majority of students (51 %) would want the videos available for download. Eighty-five percent of the overall student group rated the videos at least 4 out of 5.

More students in the DEM5/GEM4 year groups agreed or strongly agreed that the videos helped in identifying the appropriate questions when taking a history (97 % vs. 88 %, p = 0.022), and improved confidence in interpreting history and clinical examination findings (92 % vs. 79 %, p = 0.003), when compared to the other year groups. Similarly, the DEM5/GEM4 groups agreed that the videos improved preparation for exams (88%vs. 74 %, p = 0.005) and expanded exposure to surgical patients (93 % vs. 81 %, p = 0.004) compared to the other year groups. The overall experience of DEM5/GEM4 students was higher than that of the other year groups (p = 0.029) (Table 3).

**Table 3:** Differences in questionnaire response when comparing the final year groups (DEM5/GEM4) and other year groups (DEM3/GEM2 & DEM4/GEM3).

Question	Answer	DEM3/GEM2 & DEM4/GEM3 N = 191 N(%)	DEM5/GEM4 N = 118 N(%)	Total N = 309 N(%)	p-Value
The recordings help to Identify appropriate questions to ask when taking a history	SD/D/N	**22 (12)**	**4 (3)**	**26 (8)**	**0.022**
	A/SA	**169 (88)**	**114 (97)**	**283 (92)**	
The recordings help to give Insight into how to perform clinical examination	SD/D/N	17 (9)	5 (4)	22 (7)	0.173
	A/SA	172 (91)	114 (96)	286 (93)	
The recordings help to improve confidence in interpreting history and examination findings	SD/D/N	**40 (21)**	**9 (8)**	**49 (16)**	**0.003**
	A/SA	**149 (79)**	**110 (92)**	**259 (84)**	
The recordings were ambiguous or confusing	SD/D/N	169 (89)	112 (95)	281 (92)	0.141
	A/SA	20 (11)	6 (5)	26 (9)	
The text slides were useful to reinforce key points	SD/D/N	24 (13)	14 (12)	38 (12)	0.962
	A/SA	166 (87)	105 (88)	271 (88)	
The text slides are useful in giving additional detail to difficult concepts	SD/D/N	39 (21)	17 (14)	56 (18)	0.209
	A/SA	150 (79)	102 (86)	252 (82)	
The text slides distracted from the clinical video	SD/D/N	171 (91)	111 (93)	282 (92)	0.515
	A/SA	18 (9)	8 (7)	26 (8)	
The recordings Improved my preparedness for exams	SD/D/N	**49 (26)**	**14 (12)**	**63 (21)**	**0.005**
	A/SA	**141 (74)**	**104 (88)**	**245 (79)**	
The recordings were useful in expanding my exposure to surgical patients	SD/D/N	**36 (19)**	**8 (7)**	**44 (14)**	**0.004**
	A/SA	**151 (81)**	**111 (93)**	**262 (86)**	
The recordings should include quiz/self-assessment	SD/D/N	43 (23)	38 (32)	81 (26)	0.105
	A/SA	145 (77)	81 (68)	226 (74)	
The recordings should include more detail regarding investigation & management	SD/D/N	49 (26)	31 (26)	80 (26)	1.000
	A/SA	139 (74)	88 (74)	227 (74)	
The recordings should be available for download	SD/D/N	76 (40)	62 (52)	138 (45)	0.059
	A/SA	112 (60)	57 (48)	169 (55)	
The recordings should include more explicit learning objectives	SD/D/N	112 (60)	84 (71)	196 (64)	0.067
	A/SA	76 (40)	35 (29)	111 (36)	
Rate the overall experience	1, 2, 3	28 (15)	**7 (6)**	**35 (12)**	**0.029**
	4, 5	156 (85)	**107 (94)**	**263 (88)**	

SD/D/N- Strongly Disagree/Disagree/Neutral, A/SA- Agree/Strongly Agree. Boldface indicates statistical significance.

Other online resources used by students included; Geeky Medics (https://geekymedics.com/), Teach Me Surgery (https://teachmesurgery.com/), OSCE Stop (https://oscestop.education/), and searching other educational material on YouTube (https://www.youtube.com/). Other resources used by students included Alisdair Scott Surgery notes, Stanford University note, or the University of Leicester notes.

## Discussion

Surgery is a core component of every medical students' training. However, given the limitations of the calendar year and the commitments to fulfil coursework in other specialties, the exposure to surgical patients is inherently limited. The lack of structure during surgical rotations has previously been noted [4].

This online compendium of surgical education videos was created to supplement the education of medical students in the authors' institution. Each of the educational videos consisted of a consultant surgeon taking a history and/or performing clinical examination with a set of additional explanatory notes. Although this resource was in development prior to the COVID 19 pandemic, it became a principal component of surgical education, at times for whole year groups excluded from the hospital environment and at other times for isolating individual medical students. The feedback from the students across all three student year groups was predominantly positive, highlighting the major role that a novel education tool, such as real patient recordings, can have for medical students.

Clinical medical education is typically delivered through different media including textbooks, lectures, and bedside teaching. In our medical school, students are given a list of recommended textbooks at the start of the academic year. Although textbooks are a valuable resource and reference for students, high yield condensed resources are important to students given the time commitments with clinical attachments. With this series of video recordings, students get the opportunity to see real patients being assessed and examined by a consultant. This helps to broaden students' exposure to patients that they may not have seen on rotations, due to time limitations. The accompanying notes are valued by students firstly as an aid to interpretation of the patient's history and clinical examination findings; secondly as an example of how verbal communications might be translated into written notes for the patient records and thirdly; to add clarity to complex issues.

The use of e-learning needs to be carefully designed and integrated into medical education. This video compendium was developed in a planned manner to encompass the surgical curriculum. In a study from an American University, an eLearning program was developed during the pandemic, but the medical student group that was surveyed felt that it was too rapidly set up and was fragmented in its content. Interestingly the students expressed a desire for real cases to follow [1]. This is a considerable advantage of the real patient video compendium, which helps to bridge some of the limitations of current e-learning platforms.

A considerable proportion of students had a positive experience with the video compendium and this effect spanned across year groups. Final year medical students are not only preparing for real patients interaction in their end of year examinations, but also for real patient interactions in the workplace. The other year groups have OSCEs, with role players in their end of year “clinical examination.” Not unexpectedly, the final year groups had a strong positive experience of the video compendium compared to the other year groups. Interestingly, a large proportion of students watched the videos on a number of occasions, supporting their use as a revision tool. A similar learning platform was created for paediatrics in another Irish university that encompassed online lectures and scenarios, clinical examination videos, discussion forums and video conferencing tutorials [5]. This, too, was well received by students and was held to be a very beneficial resource to prepare for exams and for clinical practice. A previous web-based platform for problem based learning was developed in our institution and was found to improve the examination results of final year medical students in our university [6].

Given the success of this education tool with medical students, there is significant potential for the videos to be made available to post graduate general practice and surgical trainees. The videos were exclusively available on the university e-learning platform, but there is potential for it to be shared among other universities. The results of the survey suggest that a recorded real patient interaction has compelling benefit to medical student education and this approach could be broadened out to other specialties.

The following are the supplementary data related to this article.

Supplementary Table 1 List of questions from final Questionnaire. Supplementary Table 1

Supplementary Table 2 Student use of accompanying tutorials and quizzes. Supplementary Table 2

## Funding sources

There is no funding source to declare.

## Ethics approval

Ethical approval was granted by the Clinical Research Ethics Committee of the Cork Teaching Hospitals.

## Funding statement

There was no external funding for this study.

## CRediT authorship contribution statement

**Stephen J. O'Brien:** Methodology, Formal analysis, Data curation, Writing – original draft, Writing – review & editing. **Michelle Reardon:** Conceptualization, Methodology, Resources, Writing – original draft, Writing – review & editing, Supervision. **Gerald McGreal:** Conceptualization, Methodology, Resources, Writing – original draft, Writing – review & editing, Supervision.

## Declaration of competing interest

The authors declare no conflict of interest.
