# Supplementary material for: A novel video compendium of real surgical patient interactions for medical students
Source: Surg Open Sci. 2023 Jul 27;14:128–34. doi: 10.1016/j.sopen.2023.07.017 (PMC10436175; doi:10.1016/j.sopen.2023.07.017)
Supplement: Supplementary Table 1 — List of questions from final Questionnaire. [file mmc1.docx]

**Supplementary Table 1**. List of questions from final Questionnaire

| **Question** | **Stem** | **Answer** |
| --- | --- | --- |
| **1** | What is your age? | Free text |
| **2** | What is your nationality? | Free text |
| **3** | Which programme are you studying? | Direct Entry Medicine/ Graduate Entry medicine |
| **4** | What is your gender? | Free text |
| **5** | Was your experience of Surgical Panopto tutorials and patient videos hindered by visual/auditory/technology issues? (Explain briefly if so). | Free text |
| **6** | What impact did COVID have on your HOSPITAL placement? | No impact/1 week missed/ 2 weeks missed/ >2 weeks missed |
| **7** | What proportion of the Panopto patient recordings of Mr McGreal interviewing and examining patients did you complete? | <10/ 10-20/ 20-30/ 30-40 |
| **8** | The Panopto Patient Recordings of Mr McGreal interviewing and examining patients; were valuable in helping me to identify APPROPRIATE QUESTIONS TO ASK when taking a history. | SD/ D/N/A/SA |
| **9** | The Panopto Patient Recordings of Mr McGreal interviewing and examining patients were valuable insight into how to COMMUNICATE well with patients | SD/ D/N/A/SA |
| **10** | The Panopto Patient Recordings of Mr McGreal interviewing and examining patients were valuable insight into how to PERFORM CLINICAL EXAMINATION | SD/ D/N/A/SA |
| **11** | The Panopto Patient Recordings of Mr McGreal interviewing and examining patients improved my understanding of HOW TO ARTICULATE FINDINGS e.g.describing what is seen on inspection | SD/ D/N/A/SA |
| **12** | The Panopto Patient Recordings of Mr McGreal interviewing and examining patients improved my confidence in INTERPRETING history and clinical examination findings | SD/ D/N/A/SA |
| **13** | The Panopto Patient Recordings of Mr McGreal interviewing and examining patients were often ambiguous or confusing | SD/ D/N/A/SA |
| **14** | The TEXT SLIDES accompanying Panopto Patient Recordings of Mr McGreal interviewing and examining patients; were useful to REINFORCE KEY POINTS | SD/ D/N/A/SA |
| **15** | The TEXT SLIDES accompanying Panopto Patient Recordings of Mr McGreal interviewing and examining patients; were useful to REINFORCE the ORGANISATION/STRUCTURE/SEQUENCE in history/clinical exam | SD/ D/N/A/SA |
| **16** | The TEXT SLIDES accompanying The Panopto Patient Recordings of Mr McGreal interviewing and examining patients; were useful in giving ADDITIONAL DETAIL to explain difficult concepts WHEN NEEDED | SD/ D/N/A/SA |
| **17** | The TEXT SLIDES accompanying the the Panopto Patient Recordings of Mr McGreal interviewing and examining patients ADDED CLARITY | SD/ D/N/A/SA |
| **18** | The TEXT SLIDES accompanying the the Panopto Patient Recordings of Mr McGreal interviewing and examining patients DISTRACTED FROM THE CLINICAL VIDEO | SD/ D/N/A/SA |
| **19** | If you found the text slides unhelpful or distracting please expand by adding a comment e.g. if the clinical image became a small window that you didn't know how to swap into the main screen | Free text |
| **20** | The Panopto Patient Recordings of Mr McGreal interviewing and examining patients CAPTURED AND MAINTAINED my ATTENTION | SD/ D/N/A/SA |
| **21** | The Panopto Patient Recordings of Mr McGreal interviewing and examining patients IMPROVED MY CONFIDENCE WITH PATIENTS | SD/ D/N/A/SA |
| **22** | The Panopto Patient Recordings of Mr McGreal interviewing and examining patients IMPROVED MY PREPAREDNESS FOR EXAMS | SD/ D/N/A/SA |
| **23** | The Panopto Patient Recordings of Mr McGreal interviewing and examining patients were EFFICIENT and SATISFYING in achieving learning objectives | SD/ D/N/A/SA |
| **24** | The Panopto Patient Recordings of Mr McGreal interviewing and examining patients were A SOURCE OF FRUSTRATION TO ME | SD/ D/N/A/SA |
| **25** | If the recordings of Mr McGreal interviewing and examining patients were A SOURCE OF FRUSTRATION to you, please explain why? | Free text |
| **26** | Did you search for other videos on-line of clinicians interviewing and examining patients/role players? | Free text |
| **27** | The on-line RECORDINGS OF CLINICIANS TAKING A HISTORY OR PERFORMING CLINICAL EXAMINATION that I found most useful were found through (e.g. Geeky Medics, Teach me Surgery); | Free text |
| **28** | In what way were the RECORDINGS OF CLINICIANS TAKING A HISTORY OR PERFORMING CLINICAL EXAMINATION that you found IN YOUR OWN ONLINE SEARCHES more useful than the Panopto recordings of Mr McGreal with patients? | Free text |
| **29** | The Panopto Patient Recordings of Mr McGreal interviewing and examining patients was useful in expanding my exposure to SURGICAL patients? | SD/ D/N/A/SA |
| **30** | WITHOUT The Panopto Patient Recordings of Mr McGreal interviewing and examining patients- I would feel inadequately prepared to progress to surgical internship next year | SD/ D/N/A/SA |
| **31** | The Panopto Patient Recordings of Mr McGreal interviewing and examining patients; should be compulsory content for third/fourth/final year medical students | SD/ D/N/A/SA |
| **32** | The Panopto Patient Recordings of Mr McGreal interviewing and examining patients should include quiz/self-assessment | SD/ D/N/A/SA |
| **33** | The Panopto Patient Recordings of Mr McGreal interviewing and examining patients should include more detail regarding investigation & management | SD/ D/N/A/SA |
| **34** | The Panopto Patient Recordings of Mr McGreal interviewing and examining patients should be better aligned and complemented by related teaching material | SD/ D/N/A/SA |
| **35** | The Panopto Patient Recordings of Mr McGreal interviewing and examining patients should be easier to find on Canvas | SD/ D/N/A/SA |
| **36** | The Panopto Patient Recordings of Mr McGreal interviewing and examining patients should be available for download | SD/ D/N/A/SA |
| **37** | The Panopto Patient Recordings of Mr McGreal interviewing and examining patients should include more explicit learning objectives | SD/ D/N/A/SA |
| **38** | Rate the overall value to you of  The Panopto Patient Recordings of Mr McGreal interviewing and examining patients | 1 star- 5 stars |
| **39** | Any other comments you would like to add? | Free text |

SD- Strongly Disagree, D- Disagree, N- Neutral, A- Agree, SA- Strongly Agree
