# Supplementary material for: A novel video compendium of real surgical patient interactions for medical students
Source: Surg Open Sci. 2023 Jul 27;14:128–34. doi: 10.1016/j.sopen.2023.07.017 (PMC10436175; doi:10.1016/j.sopen.2023.07.017)
Supplement: Supplementary Table 2 — Student use of accompanying tutorials and quizzes. [file mmc2.docx]

Supplementary Table 2. Student use of accompanying tutorials and quizzes

|  | 4^th^ DEM/3^rd^ GEM  N=172  N (%) | 5^th^ DEM/4^th^ GEM  N=206  N (%) |
| --- | --- | --- |
| **Tutorials** |  |  |
| Abdominal Aortic Aneurysm | 44(26) | 42(20) |
| Carotid disease | 38(22) | 32(16) |
| Diabetic Vascular | 36(21) | 36(17) |
| Gastrointestinal tract Surgery | 53(31) | 107(52) |
| Leg Ulcer | 17(10) | 31(15) |
| Peripheral vascular occlusive disease | 46(27) | 49(24) |
| Venous Hypertension | 24(14) | 29(14) |
|  |  |  |
| **Quizzes** |  |  |
| Peri-operative Care | 79(46) | 29(14) |
| Post-operative Pyrexia | 54(31) | 15(7) |
| Pot-operative Paralytic Ileus | 52(30) | 14(7) |
| Bleeding and Urinary Retention | 43(25) | 12(6) |
| Diabetic Foot & Peri-operative Care | 76(44) | 25(12) |
| Fluids | 68(40) | 28(14) |
| Cramping Leg | 17(10) | 10(5) |

DEM- Direct Entry Medicine, GEM- Graduate Entry Medicine
